# Genetic Analysis of Ligation-Induced Neointima Formation in an F2 Intercross of C57BL/6 and FVB/N Inbred Mouse Strains

**DOI:** 10.1371/journal.pone.0121899

**Published:** 2015-04-13

**Authors:** Caroline Östergren, Jeong Shim, Jens Vinther Larsen, Lars Bo Nielsen, Jacob F. Bentzon

**Affiliations:** 1 Department of Clinical Medicine, Aarhus University, and Department of Cardiology, Aarhus University Hospital, Aarhus, Denmark; 2 Department of Clinical Biochemistry, Rigshospitalet, University of Copenhagen, Copenhagen, Denmark; 3 Department of Biomedical Sciences and Department of Clinical Medicine, University of Copenhagen, Copenhagen, Denmark; University of Iowa, UNITED STATES

## Abstract

**Objective:**

Proliferation and migration of vascular smooth muscle cells (SMCs) are central for arterial diseases including atherosclerosis and restenosis. We hypothesized that the underlying mechanisms may be modeled by carotid ligation in mice. In FVB/N inbred mice, ligation leads to abundant neointima formation with proliferating media-derived SMCs, whereas in C57BL/6 mice hardly any neointima is formed. In the present study, we aimed to identify the chromosomal location of the causative gene variants in an F2 intercross between these two mouse strains.

**Methods and Results:**

The neointimal cross-sectional area was significantly different between FVB/N, C57BL/6 and F1 female mice 4 weeks after ligation. Carotid artery ligation and a genome scan using 800 informative SNP markers were then performed in 157 female F2 mice. Using quantitative trait loci (QTL) analysis, we identified suggestive, but no genome-wide significant, QTLs on chromosomes 7 and 12 for neointimal cross-sectional area and on chromosome 14 for media area. Further analysis of the cross revealed 4 QTLs for plasma cholesterol, which combined explained 69% of the variation among F2 mice.

**Conclusions:**

We identified suggestive QTLs for neointima and media area after carotid ligation in an intercross of FVB/N and C57BL/6 mice, but none that reached genome-wide significance indicating a complex genetic architecture of the traits. Genome-wide significant QTLs for total cholesterol levels were identified on chromosomes 1, 3, 9, and 12.

## Introduction

Phenotypic modulation, migration and proliferation of smooth muscle cells (SMCs) play central roles in the development of both atherosclerotic lesions and restenosis after percutaneous coronary intervention (PCI). In atherosclerosis, SMCs modulate to a synthetic phenotype and produce fibrous tissue that if abundant can cause chronic stenoses and stable angina, but also forms the fibrous cap of plaques that protects against plaque rupture and thus acute coronary syndromes [1,2]. Following PCI, synthetic-type SMCs repair the vessel injury, but the response may be exaggerated leading to renewed stenosis and the need for further intervention [3]. The use of drug-eluting stents has substantially reduced, although not eradicated, this problem, but also entails more intensive dual antiplatelet therapy to prevent stent thrombosis [3,4].

The recruitment of SMCs to neointimal lesions after PCI in humans is partly under genetic control [3,5,6]. Similarly, neointimal hyperplasia after different forms of vascular injury or flow-cessation in mice has been found to be highly strain dependent although little is known about the causative gene variants [7,8]. A particularly pronounced difference is seen between FVB/N inbred mice that form abundant neointima after carotid artery ligation, and C57BL/6 (B6) inbred mice that hardly form any [8]. In the present study, we aimed to identify the chromosomal locations of genes involved in the recruitment of SMCs to vascular lesions using quantitative trait locus (QTL) analysis of an F2 intercross between these two mouse strains. Furthermore, we analyzed for QTLs affecting total cholesterol levels in the cross.

## Methods

### Animals

Procedures were approved by the Danish Animal Experiments Inspectorate (2007/561–1347). At Taconic M&B Ry, female FVB/N (FVB/NTac) and male B6 (C57BL/6JBomTac) mice where crossed to form F1 hybrids and these where again intercrossed to obtain F2 mice. Female mice, 9–10 weeks old, were used for the experiments. For all surgical procedures, mice where anesthetized with isoflurane (induction 5%, maintenance 1.5–2.0%) and buprenorphine (0.1 mg/kg s.c) and postoperative analgesic (Rimadyl, 5 mg/kg s.c) where given every 24 hours for 3 days.

### Carotid artery ligation

The neck was incised through the midline, and the left common carotid artery was dissected free of surrounding tissue and ligated near the carotid bifurcation using a 6–0 silk ligature as described [9]. Four weeks after the ligation, the mice were anaesthetized (pentobarbital 5 mg i.p. in 2% lidocain) and killed by exsanguination. Spleens were removed and snap-frozen in liquid nitrogen. Then, the mice were flushed with a cardioplegic solution, perfusion-fixed at ~100 mmHg with 4% phosphate-buffered formaldehyde (pH 7.2) via the left ventricle and then immersed in the fixative for 24 hours before storage in cold phosphate buffer. The left common carotid artery was removed, embedded in paraffin, sectioned at 3 μm thickness and stained with orcein for morphometric analysis.

### Morphometric analysis

Neointima and media areas were analyzed using computer–assisted image analysis (Image J, National Institutes of Health). The lumen area and the areas encompassed by the internal (IEL area) and external (EEL area) elastic laminae were first measured. Neointima area was then calculated as IEL area—lumen area, and media area as EEL area—IEL area. The intima-media ratio was calculated as neointima area/media area.

Cross-sections from parental B6 and FVB/N strains obtained at 1, 2, 3, and 4 mm proximal to the ligature were analyzed in pilot studies to determine the most appropriate site for analysis in F2 mice. Analysis at the 4 mm level showed the highest difference in neointima formation between strains, whereas sections closer to the ligature frequently contained signs of thrombus. Thus, the 4 mm section was chosen for final analysis of parental strains as well as F1 and F2 mice.

### Plasma analysis

Non-fasting EDTA-plasma samples from B6 and FVB/N mice at 8–10 weeks of age were obtained from Jackson Laboratories (Bar Harbor, Maine, USA). Non-fasting EDTA-plasma samples from F2 mice were collected at the time of euthanization. Total cholesterol was measured manually, in duplicate, with an enzymatic cholesterol reagent (CHOD-PAP, Roche Diagnostics).

Hepatic lipase activity was measured using the Confluolip kit (Progen, Heidelberg, Germany) in EDTA-plasma, which has previously been shown to be appropriate because virtually all hepatic lipase in mice is present in an unbound form [10].

### Genome scan and QTL analysis

DNA was prepared from spleens using DNeasy Blood and Tissue Kit (Qiagen, Denmark) and genotyped for single-nucleotide polymorphisms (SNPs) on Illuminas mouse medium-density linkage panel. The genotyping was performed with the use of Illuminas Golden Gate Assay and BeadStation by AROS Applied Biotechnology A/S (Aarhus, Denmark). The mouse medium-density linkage panel consists of 1449 SNPs covering the entire mouse genome. Each SNP allele was verified using DNA from 3 FVB/N mice, 3 B6 mice and 3 F1 mice. Loci with no variation between B6 and FVB/N mice and SNPs that did not cluster clearly in AA (FVB/N), AB (heterozygote) and BB (B6) genotype calls with the expected distribution were excluded.

The QTL analysis was performed in the R/qtl package in R [11]. The centimorgan (cM) position of SNPs were taken from the sex-averaged (for autosomes) and female-specific (for the X chromosome) revised mouse genetic map [12]. The physical (megabase (Mb)) positions (GRCm38) of the QTLs and their intervals were obtained using the Map Converter tool (http://cgd.jax.org/mousemapconverter/). Genome scans for neointima and intima-media ratio were performed by non-parametric interval mapping to calculate LOD scores (log of the odds ratio) at 1 cM intervals. Media areas were log transformed to follow a normal distribution and analyzed by parametric interval mapping. Confidence intervals (95%) for the LOD peak scores were calculated in R/qtl using the Bayesian credible interval and expanded to the nearest marker.

QTL analysis for cholesterol levels was performed using a two-dimensional QTL scan followed by fitting genome-wide significant QTLs into an additive model. Bayesian credible intervals for the QTLs were refined using the refineqtl function of R/qtl.

Genome-wide significance thresholds were determined empirically by 10000 (for one-dimensional scans) and 1000 (for two-dimensional scans) permutations of the trait data with the genome-wide thresholds set to p<0.05 as significant, p<0.1 as highly suggestive and p<0.63 as suggestive [13].

### Statistical analysis

Statistical analysis of neointima and media data were performed in GraphPad Prism 5.0 statistical software (GraphPad, San Diego, CA). Data were analyzed by non-parametric or parametric tests as indicated in text and figure legends. P<0.05 was considered to be statistically significant.

## Results

### Phenotypic analysis

**Fig 1:**
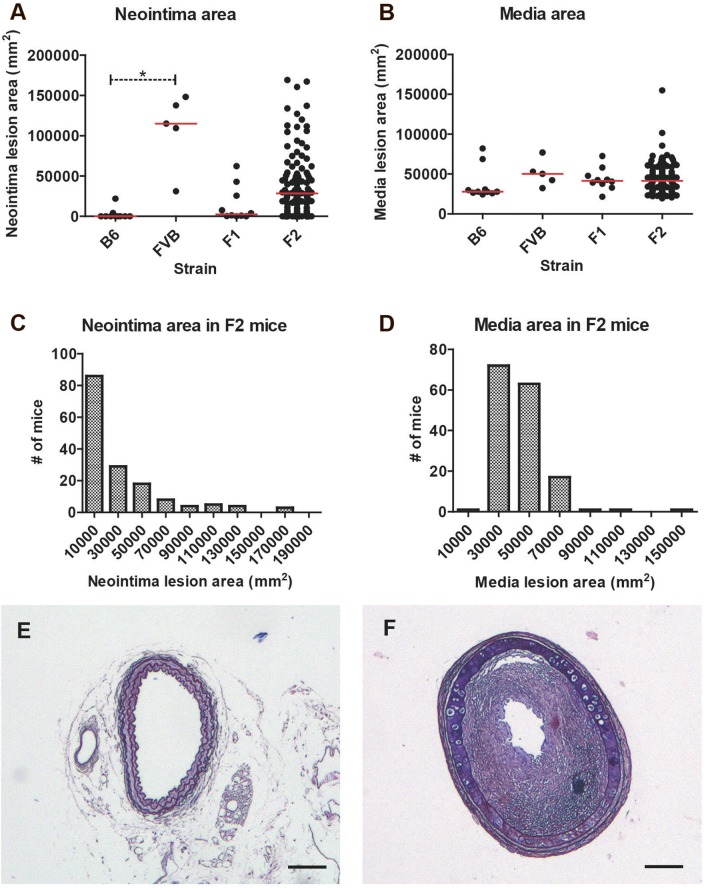
A-B. Neointima (A) and media (B) area (μm^2^) in B6 (n = 10), FVB/N (n = 5), F1 (n = 10) and F2 (n = 157) mice 4 mm proximal to the ligation after 4 weeks. The red lines represent median values. *P<0.01 by Mann-Whitney test. **C-D.** Frequency histogram for neointima (C) and media (D) area (μm^2^) in the F2 mice. **E-F.** Representative orcein-stained histological sections of common carotid arteries from B6 (E) and FVB (F) mice. The section is taken 4 mm proximal to ligature. Scale bars = 100 μm. Neointima areas in the shown images are 0 μm^2^ (E) and 115131 μm^2^ (F) and media area 27829 μm^2^ and 52998 μm^2^, respectively.

Four weeks after carotid ligation, FVB/N inbred mice had much larger neointimal lesion area than the B6 inbred mice (**Fig 1A**) in consistence with previous reports.^9^ The median media area was also larger in FVB mice than B6 mice although not statistically significant (**Fig 1B**). For both traits, F1 and F2 mice showed intermediate median levels. Neointima areas in the F2 mice were frequency plotted and showed a reverse J-shaped distribution, while media areas followed a log-normal distribution (**Fig 1C and 1D**). A significant, but modest positive correlation (r^2^ = 0.20, p<0.0001, linear regression, S1 Fig) was found between neointima and media area. Examples of histological sections of common carotid arteries from B6 and FVB/N mice are shown in **Fig 1E and 1F**.

### QTL analysis for neointima

**Fig 2:**
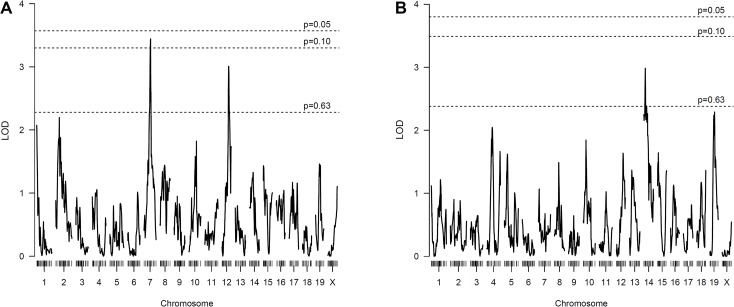
Whole genome scan LOD score plot for neointima (A) and media (B) area in the F2 mice, showing significance thresholds.

**Fig 3:**
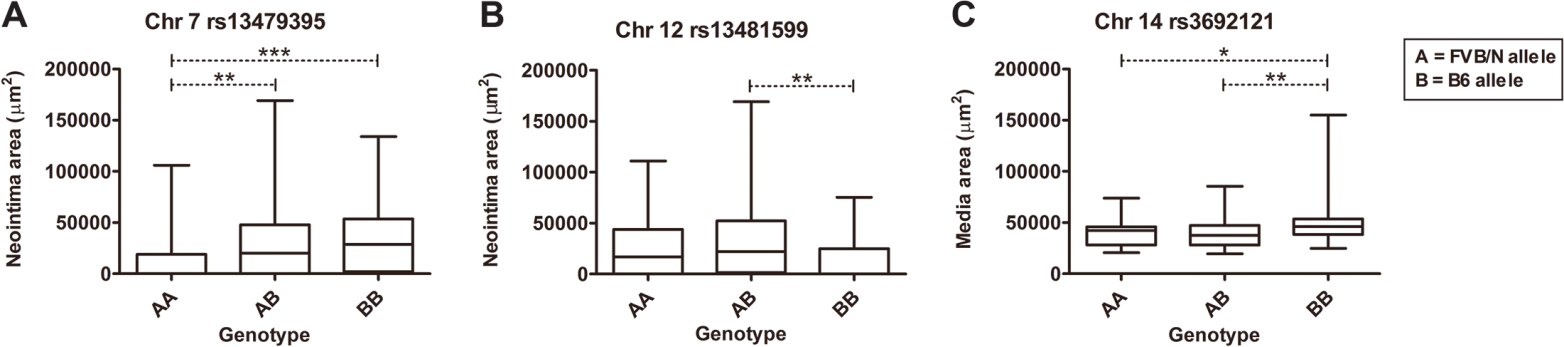
Allelic contribution to neointima lesion area at the putative QTLs. A and B represent FVB/N and B6 alleles, respectively. **A.** For the putative QTL on chromosome 7 at rs13479395, the AA genotype had a significantly smaller lesion area than both the AB and BB genotypes, while there was no significant difference between the two genotypes AB and BB. **B.** For the putative QTL on chromosome 12 at rs13481599, the lesion areas in the F2 mice with the BB genotype were significantly smaller than for the AB and AA genotypes. **C.** For the putative QTL for media area on chromosome 14 at rs3692121, larger media areas were seen in mice with the BB genotype. Box-plots show median (middle bar), 25% and 75% percentiles (bottom and top of box), and range (whiskers). *P<0.05, **p<0.01, ***p<0.001 by Kruskal-Wallis with Dunn’s post-test (A and B) or ANOVA with Tukey’s post-test (C).

We found 800 SNPs that were clearly genotyped and different between B6 and FVB/N mice and used these to conduct genome scans in 157 female F2 mice for neointima area, media area, and intima-media ratio. This identified two suggestive QTLs for neointima formation (**Table 1** and **Fig 2**), but none that reached genome-wide significance. A highly suggestive QTL was on chromosome 7 (LOD = 3.44, p = 0.067) with a peak LOD score near the marker rs13479395 and accounting for 6.99% of the phenotypic variance in the F2 population. Paradoxically, the F2 mice with both alleles from the B6 parental strain (BB) at this locus had the largest neointima lesion area (**Fig 3A**). Another suggestive QTL was on chromosome 12 (LOD = 3.01, p = 0.196) with a peak LOD score near the marker rs13481599 and accounting for 7.1% of the variance. At this locus, F2 mice that were homozygous and heterozygous for the FVB/N allele (AA and AB) had larger lesion areas than those with only B6 alleles (BB) (**Fig 3B**). The Bayesian credible interval for the LOD peak score for the putative QTL on chromosome 7 (33.06–58.74 cM or 53.09–109.72 Mb) contains 573 protein-coding genes. On chromosome 12, the confidence interval for the LOD peak score (38.88–62.65 cM or 83.78–116.72 Mb) contains 284 protein-coding genes.

**Table 1:** Neointima and media QTLs.

Phenotype	Chr	SNP*	Peak position cM† (Mb‡)	CI cM† (Mb‡)	LOD score	p-value	High allele	# of genesǂ in CI
Neointimaarea	7	rs13479395	50.46 (90.02)	33.06–58.74 (53.09–109.72)	3.44	0.067	B6	573
Neointima area	12	rs13481599	46.73 (96.66)	38.88–62.65 (83.78–116.72)	3.01	0.196	FVB	284
Media area	14	rs3692121	13.70 (23.74)	4.70–47.80 (8.06–97.14)	2.98	0.251	B6	761

* SNP marker closest to LOD peak score.

† centimorgan position of LOD peak score and interval.

‡ physical position of LOD peak score and interval.CI, 95% Bayesian credible interval.

ǂ annotated protein-coding genes in LOD peak interval. CI, 95% Bayesian credible interval.

For media area, a suggestive LOD peak was found on chromosome 14 (**Table 1**), with the B6 allele associated with the higher area, ie. opposite to the trend seen in parental strains. The confidence interval was broad, however, encompassing more than 700 protein-coding genes. Scans for intima-media ratio yielded results that were highly similar to those for neointima formation (data not shown).

### QTL analysis for total cholesterol

Mild modulations of plasma cholesterol levels with high-fat feeding have been shown to affect the development of ligation-induced neointima in B6 mice indicating that lipoprotein levels may modulate the recruitment of SMCs to the intima [14]. Non-fasting total cholesterol was different in the FVB/N (4.15±0.51 mM) and B6 (2.37±0.35 mM) parental strains, and the presence of segregating genes affecting plasma cholesterol in the F2 population thus offered a possibility to look for effects of plasma cholesterol on neointima formation. No correlation, however, was found between the traits (r^2^<0.01, non-significant, linear regression, S2 Fig).

**Fig 4:**
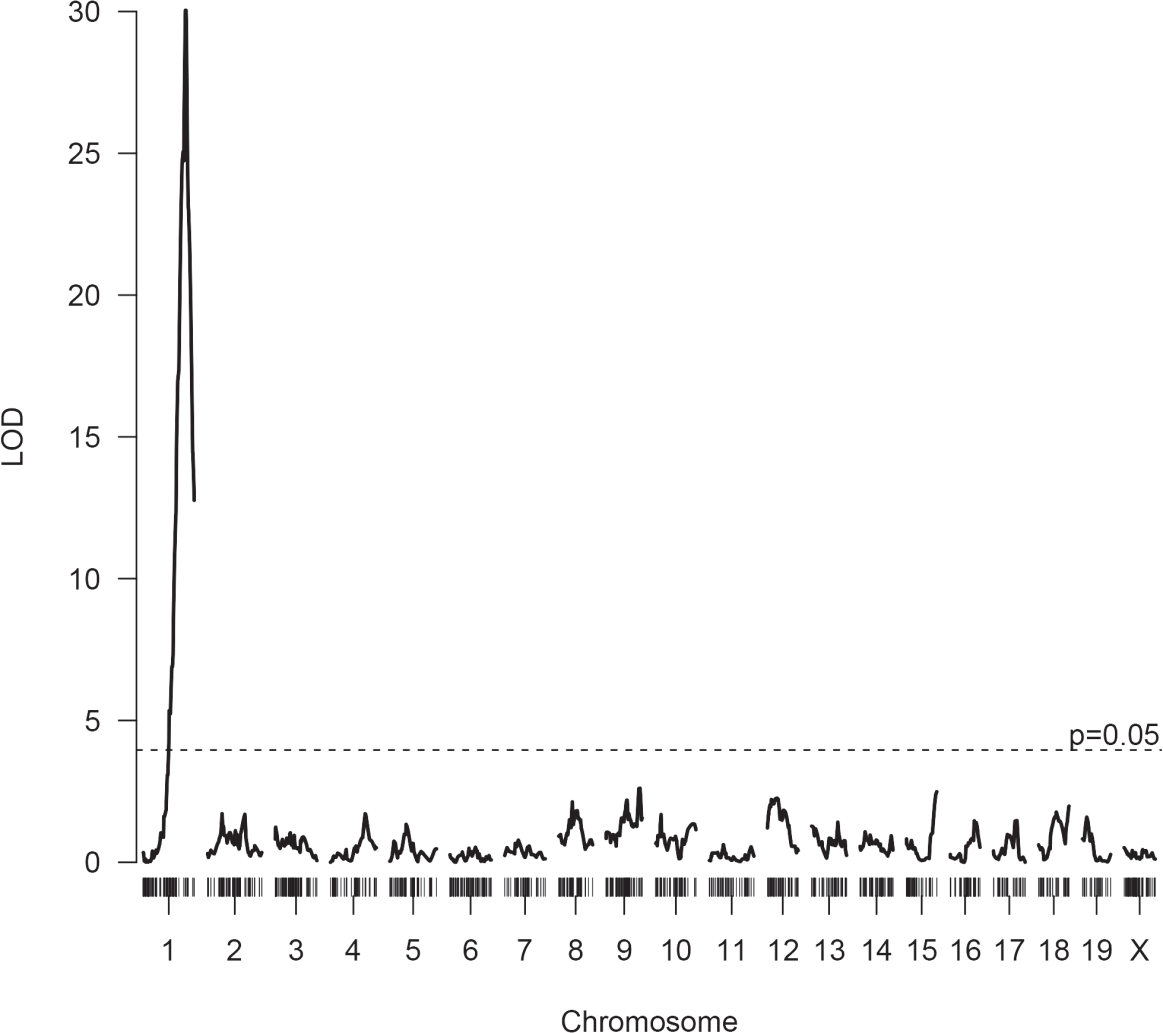
Whole genome scan LOD score plot for plasma total cholesterol in the F2 mice showing significance thresholds.

**Fig 5:**
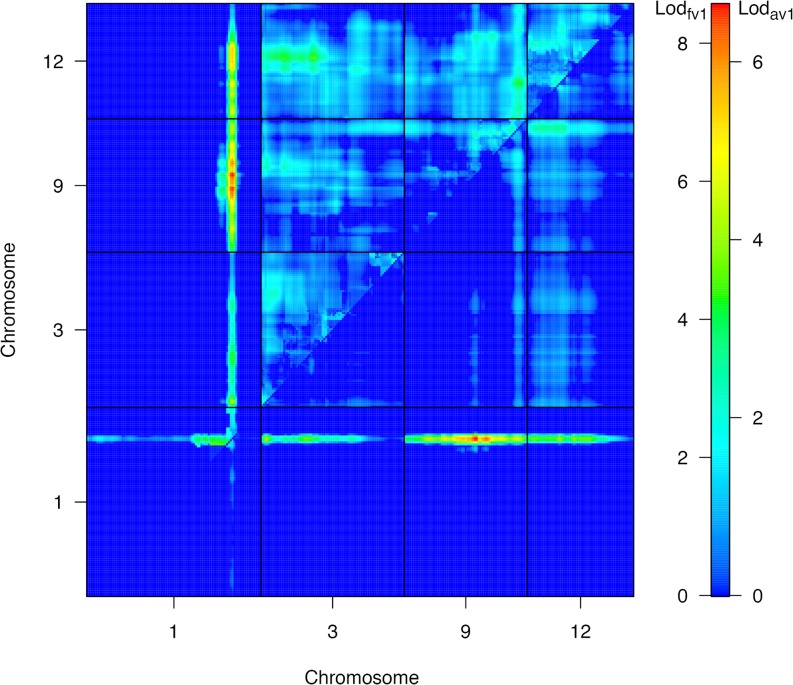
Two-dimensional QTL scan for plasma total cholesterol levels. The upper left triangle shows LOD_fv1_ scores indicating the likelihood of a two-QTL model allowing for epistasis. The lower right triangle shows LOD_av1_ indicating the likelihood of an additive QTL model without epistasis. There is evidence for a second QTL on chromosomes 3, 9, 12 in extent to that on distal chromosome 1 with little indication of epistasis. Numbers to the left and right on the color scale corresponds to LOD_fv1_ and LOD_av1_ scores, respectively.

Although unrelated to the neointima trait, we took advantage of the F2 cross to further explore genes involved in regulation of plasma total cholesterol. A one-dimensional QTL scan of the F2 cross revealed a strong QTL on distal chromosome 1 with a LOD peak score of 30.0, which was consistent with the difference in the genotype in the *Apoa2* locus between these mouse strains (*Apoa2* ^*b*^ in FVB/N versus *Apoa2* ^*a*^ in B6) previously reported as a QTL in several mouse crosses (**Fig 4**) [15]. To search for additional QTLs that may be masked by the strong effect of the chromosome 1 QTL, we conducted a two-dimensional QTL scan and found evidence for three additive QTLs at chromosome 3, 9, and 12 (**Table 2**) (**Fig 5**). Fitting the four QTLs into an additive model, we find that the QTL on chromosome 1 explained 59.8% of variance in plasma cholesterol, while the other QTLs on chromosomes 3, 9, and 12 explained 3.2%, 4.3% and 1.9%, respectively (**Table 2**).

**Table 2:** Cholesterol QTLs.

Chr	SNP*	Position cM† (Mb‡)	CI cM† (Mb‡)	LOD score	p-value**	variance %	high allele
1	rs3669108	81.65 (177.58)	76.73–83.62 (169.31–179.82)	38.72	<2 10^–16^	59.76	FVB
3	gnf03.015.035	32.15 (69.01)	4.04–60.27 (16.45–130.37)	3.65	3.6 10^–4^	3.192	B6
9	rs13480365	51.01 (97.29)	32.40–59.63 (59.99–109.26)	4.85	2.7 10^–5^	4.325	B6
12	rs13481527	30.30 (72.89)	5.58–46.73 (11.61–97.21)	2.24	7.8 10^–3^	1.915	B6

*SNP marker closest to LOD peak score.

**Point-wise p-value (F test) for including the QTL in the model.

†centimorgan position of LOD peak score and interval.

‡Megabase position of LOD peak score and interval.

The QTL interval on chromosome 9 includes the hepatic lipase gene *Lipc* identified in human GWAS as a potential plasma total, LDL and HDL cholesterol-modulating gene [16]. Analysis of plasma hepatic lipase activity revealed higher activity in FVB/N compared to B6 mice (33.5±2.6 vs. 28.5±3.1 pmol ml^-1^ min^-1^, p = 0.001, t-test), but similar analysis of F2 mice with the AA and BB genotype in the *Lipc* locus indicated that this difference was not controlled by genetic variation at the *Lipc* locus (data not shown). Two other candidate genes, *Msl1* and *Apoa4*, are located within or just outside the 95% CI for the chromosome 9 QTL. Expression levels of these were measured using qRT-PCR on liver samples (*Msl1*) and ELISA of plasma (APOA4), respectively, but also failed to show cis-regulation (data not shown). Furthermore, the coding sequence difference in the *Apoa4*, previously reported among other mouse strains [17], does not exist between B6 and FVB/N strains.

## Discussion

The migration of SMCs to vascular lesions plays an important role in both atherosclerosis and restenosis. It is a complex trait controlled by multiple, mostly unidentified, genes [7,8]. QTL analysis of mouse crosses is a strategy to identify some of those, and unlike the candidate gene approach it can be used to identify genes and pathways not previously known to be involved in SMC recruitment. QTL analysis has been used as the starting point to identify genes involved in many complex human diseases like liver fibrosis, atherosclerosis and type 1 diabetes [18–20].

In the present study, we performed a genome scan to identify genetic loci associated with SMC migration to vascular lesions in an F2 intercross between B6 and FVB/N mice. The neointimal response to wire injury or ligation in these strains differs particularly widely, with FVB/N mice being one of the most neointima-prone strains described and B6 one of the most neointima-resistant [8,21]. This difference was reproduced in the present study, and we identified suggestive QTLs on chromosome 7 and 12 for the trait, but none that reached genome-wide significance as defined by permutation tests. Each of the suggestive QTL was estimated to explain less than 10% of the variation indicating that the difference in neointima formation between FVB/N and B6 mice is controlled by a large number of genes each with a relatively small individual effect.

The allelic contribution to neointima area at the highly suggestive QTL on chromosome 7 was not the expected. F2 mice inheriting both B6 alleles had larger lesions than F2 mice inheriting both FVB/N alleles, i.e. opposite to what is observed in the parental strains. Provided that this is not a false-positive finding, it is an example of a cryptic locus; a phenomenon, which has also been observed in other QTL studies, e.g. of atherosclerosis susceptibility [22,23]. They reflect the fact that a QTL analysis detects loci that are responsible for the phenotypic variation in the F2 cohort and not necessarily loci that are responsible for the phenotypic difference between the two parental strains [24]. The B6 allele of the chromosome 7 QTL may induce migration and proliferation of SMCs, but this effect may be overshadowed in the parental strain by other B6 genes that inhibit SMC migration in the B6 parental strain. Alternatively, the B6 variant may be dependent of the presence of gene variants from the FVB/N background to exhibit its function. The QTL on chromosome 12 showed the more expected inheritance pattern with the B6 allele being associated with smaller lesion areas than the FVB/N allele.

Candidate approaches have identified multiple proteins and pathways that affect neointima formation. For example have β_3_-integrin, thrombospondin-1, MMP-2, MMP-9 and AIF-1 been found to induce neointima formation, and NOS2 to inhibit it after carotid artery ligation in mice [25–28]. None of these genes, however, map to the suggestive QTL confidence intervals on chromosomes 7 or 12.

To our knowledge, only one previous study, by Inoue et al [29], has directly examined the difference in neointima formation between B6 and FVB/N mice. They found that the ability of SMCs to migrate were significantly different between the strains. Furthermore, that sphingolipid-1-phosphate (S1P) stimulates migration of SMCs in FVB/N mice, but not in B6 mice due to different S1P-receptor subtypes on B6 and FVB/N SMCs. The B6 mice had higher amounts of S1P_2_ receptors, which inhibit migration whereas FVB/N mice had higher amounts of S1P_1_ receptors that facilitate migration. If the suggestive QTLs in our study can be confirmed, one possibility is that they harbor genes regulating S1P metabolism.

QTL analyses in other strains and species have produced significant QTLs for neointima formation. Nestor et al. studied ballon injury-induced neointima in 301 rats from an F2 cross of Brown Norway and spontaneously hypertensive rat strains, and detected several QTLs on chromosomes 3 and 6 [30]. Korchunov et al studied 132 mice from a backcross of the neointima-prone strain SJL onto the neointima-resistant C3H/F background using a partial ligation technique to induce carotid neointima [31]. Interestingly, they identified a suggestive QTL on chromosome 7 (peak LOD = 1.5 at 66 cM) with a confidence interval that overlaps with our suggestive QTL on chromosome 7 suggesting the possibility that the two QTLs may represent variations in the same gene(s).

The difference in atherosclerosis-susceptibility between FVB/N and B6 mice has been studied in some depth in the setting of apoE or LDL receptor deficiency [32–35]. Interestingly, the susceptibility for atherosclerosis is opposite to that of neointima formation with FVB/N mice being relatively resistant and B6 mice more susceptible. Although SMC recruitment is an important mechanism in atherosclerotic plaque development, atherogenesis is complex with multiple other mechanisms involved and thus the causative gene variants underlying susceptibility to atherosclerosis and ligation-induced neointima would be expected to show only modest overlap. Indeed, none of our suggestive peaks for neointima formation mapped to previously detected QTL loci for atherosclerosis susceptibility in crosses of hyperlipidemic FVB/N and B6 mice [22,34,36,37].

Multiple previous studies have mapped QTLs affecting cholesterol levels in inbred mouse strains (qtlarchive.com). Analysis of the present cross showed a very strong QTL on distal chromosome 1 as expected based on the known sequence differences of the *Apoa2* gene between B6 and FVB/N mice. The strong effect of this locus overshadowed that of other genomic sites in the one-dimensional analysis, but a two-dimensional QTL scan revealed 3 additional QTLs for total cholesterol. Although we attempted to find genes with known or suspected involvement in lipoprotein metabolism within the QTL on chromosome 9, a region that has not been thoroughly studied in mice before, we did not succeed in identifying cis-regulated genes among our candidates.

Limitations of our study include a relatively small intercross size. Although the size of our study is larger than another recent study that successfully detected genome-wide significant QTLs for neointima in mice [31], the non-normal distribution of neointima area in our F2 group and the enforced need to conduct QTL analysis using non-parametric statistics decreased power of our study. This may have contributed to the lack of detectable genome-wide significant neointima QTLs.

In conclusion, we identified two suggestive QTLs for neointima formation and one for media area in an F2 intercross between B6 and FVB/N inbred mouse strains, but none that reached the genome-wide significance level and each was estimated to explain less than 10% of the variation. In contrast, the cross revealed 4 genome-wide significant QTLs for total cholesterol levels. Overall our data suggest a complex genetic architecture of the neointima trait in FVB mice involving multiple genetic variants with small additive effects or a complex interaction of genes.

## Supporting Information

S1 Dataset Neointima and media lesion area and SNP-chip data (XLSX)

S1 Fig Neointima and media linear regression plot. (PDF)

S2 Fig Plasma cholesterol and neointima linear regression plot. (PDF)
