# Supplementary figures and images for: Genetic Analysis of Ligation-Induced Neointima Formation in an F2 Intercross of C57BL/6 and FVB/N Inbred Mouse Strains
Source: PLoS One. 2015 Apr 13;10(4):e0121899. doi: 10.1371/journal.pone.0121899 (PMC4395357; doi:10.1371/journal.pone.0121899)

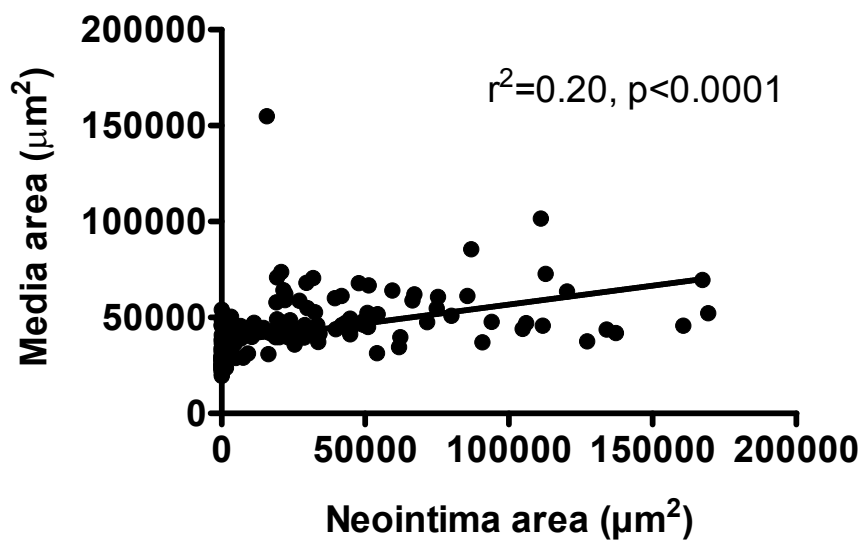

Supplement: S1 Fig — (PDF) [file pone.0121899.s002.pdf]

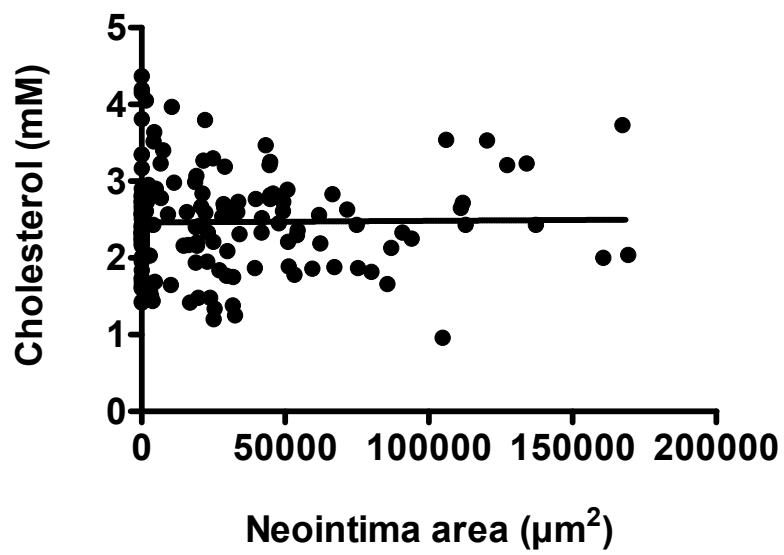

Supplement: S2 Fig — (PDF) [file pone.0121899.s003.pdf]
